# LC-MS/MS Proteomic Study of MCF-7 Cell Treated with Dox and Dox-Loaded Calcium Carbonate Nanoparticles Revealed Changes in Proteins Related to Glycolysis, Actin Signalling, and Energy Metabolism

**DOI:** 10.3390/biology10090909

**Published:** 2021-09-13

**Authors:** Hamidu Ahmed, Mokrish Ajat, Rana I. Mahmood, Rozaihan Mansor, Intan Shameha Abdul Razak, Jameel R. Al-Obaidi, Nurhanani Razali, Alhaji Zubair Jaji, Abubakar Danmaigoro, Md Zuki Abu Bakar

**Affiliations:** 1 Natural Medicines and Products Research Laboratory (NaturMeds), Institute of Bioscience, Universiti Putra Malaysia, Serdang 43400, Selangor, Malaysia; boderel748@gmail.com; 2 Department of Sciences and Engineering, Federal Polytechnic Mubi, P.M.B 35, Mubi 650221, Adamawa, Nigeria; 3 Department of Veterinary Preclinical Science, Faculty of Veterinary Medicine, Universiti Putra Malaysia, Serdang 43400, Selangor, Malaysia; mokrish@upm.edu.my (M.A.); rozaihan@upm.edu.my (R.M.); intanshameha@upm.edu.my (I.S.A.R.); jajidvm@yahoo.com (A.Z.J.); Abubaka.danmaigoro@udusok.edu.ng (A.D.); 4 Department of Biomedical Engineering, College of Engineering, Al-Nahrain University, Baghdad 64021, Iraq; rana_mahmood@eng.nahrainuniv.edu.iq; 5 Department of Biology, Faculty of Science and Mathematics, Universiti Pendidikan Sultan Idris, Tanjong Malim 35900, Perak, Malaysia; 6 Membranology Unit, Okinawa Institute of Science and Technology Graduate University, 1919-1, Tancha, Onna-son, Kunigami-kun, Okinawa 904-0495, Japan; nurhananibinti.razali@oist.jp

**Keywords:** calcium carbonate, nanoparticles, MCF-7, proteomics

## Abstract

**Simple Summary:**

This work revealed that DOX-Ar-CC-NPs have the ability to promote cell death in MCF-7 cells, showing high potency in drug delivery. DOX-Ar-CC-NPs prompts cell death of MCF-7 cancer cells in vivo. LC-MS/MS Proteomic experemnt showed alteration on the expression of proteins linked to actine signaling, carbohydrate metabolisim.

**Abstract:**

One of the most prevalent death causes among women worldwide is breast cancer. This study aimed to characterise and differentiate the proteomics profiles of breast cancer cell lines treated with Doxorubicin (DOX) and Doxorubicin-CaCO_3_-nanoparticles (DOX-Ar-CC-NPs). This study determines the therapeutic potential of doxorubicin-loaded aragonite CaCO_3_ nanoparticles using a Liquid Chromatography/Mass Spectrometry analysis. In total, 334 proteins were expressed in DOX-Ar-CC-NPs treated cells, while DOX treatment expressed only 54 proteins. Out of the 334 proteins expressed in DOX-CC-NPs treated cells, only 36 proteins showed changes in abundance, while in DOX treated cells, only 7 out of 54 proteins were differentially expressed. Most of the 30 identified proteins that are differentially expressed in DOX-CC-NPs treated cells are key enzymes that have an important role in the metabolism of carbohydrates as well as energy, including: pyruvate kinase, ATP synthase, enolase, glyceraldehyde-3-phosphate dehydrogenase, mitochondrial ADP/ATP carrier, and trypsin. Other identified proteins are structural proteins which included; Keratin, α- and β-tubulin, actin, and actinin. Additionally, one of the heat shock proteins was identified, which is Hsp90; other proteins include Annexins and Human epididymis protein 4. While the proteins identified in DOX-treated cells were tubulin alpha-1B chain and a beta chain, actin cytoplasmic 1, annexin A2, IF rod domain-containing protein, and 78 kDa glucose-regulated protein. Bioinformatics analysis revealed the predicted canonical pathways linking the signalling of the actin cytoskeleton, ILK, VEGF, BAG2, integrin and paxillin, as well as glycolysis. This research indicates that proteomic analysis is an effective technique for proteins expression associated with chemotherapy drugs on cancer tumours; this method provides the opportunity to identify treatment targets for MCF-7 cancer cells, and a liquid chromatography-mass spectrometry (LC-MS/MS) system allowed the detection of a larger number of proteins than 2-DE gel analysis, as well as proteins with maximum pIs and high molecular weight.

## 1. Introduction

Several drug delivery and drug targeting technologies are currently in use or development. Polymeric materials, microcapsules, liposomes, dendrimers, and other drug carriers all have one thing in common: they improve drug bioavailability and aggregation at the pathological site. This is extremely significant for anticancer drugs, which should specifically deliver locally with minor or no effect on the normal tissue.

Drug-containing nanoparticles can be allowed to degrade slowly, enabling the payload to be delivered at a controlled rate. Moreover, nanocarriers are suitable for delivering poorly water-soluble agents to the desired location [1]. Such factors can have a significant impact on drug efficacy and clinical acceptance. Anti-cancer drugs can be protected from biodegradation or evaporation by using nanocarriers [2].

Calcium carbonate CaCO_3_ (CC) from cockle shells have inorganic biomaterial used in the manufacturing of nanoparticles for various therapeutic purposes [3]. CaCO_3_, with a history of use in medical and pharmaceutical applications, occurs in three different forms: aragonite, calcite, and vaterite. CaCO_3_ nanoparticles (NPs) have been confirmed to be effective drug carriers for insulin [4], erythropoietin, betamethasone phosphate, and granulocyte colony-stimulating factor (G-CSF) [5], validamycin [6], gentamicin sulfate [7], and doxorubicin hydrochloride [8]. CaCO_3_ have medical criteria such as biodegradability and osteoconductivity [5,6,9].

Breast cancer is the most prevalent type of cancer affecting women and the second overall. The sustained drug delivery to the target site of the cancerous cell by a nanocarrier system improves the effectiveness of the drug. Approaches based on nanotechnology that is used to support clinical improvement from a disease often aid in understanding the interaction between cancerous cells and their microenvironment. Nanoparticles carriers have been shown to have higher loading ability, low level of toxicity, and stability of the active compound in comparison to conventional chemotherapeutic treatment [9].

Besides innovative early stage diagnosis [10], the aim to limit cancer-related deaths can also be achieved by the combination of new and effective techniques in functional genomics and proteomics, as well as bioinformatics techniques, as it has been achieved both in fundamental discoveries and clinical practice [11].

Advances in molecular genetics, especially proteomics, have opened the way for better drug development and clinical trial protocols [12]. Furthermore, proteomics provides solutions for analysing abnormal molecular alteration in cancerous cells, as well as new perspectives into creating new reagents for comprehending all stages of cancer circumstances [13]. Proteomics also offers methods for drug exploration [14].

The detection of possible proteins in breast cancer patients can help in early diagnosis, treatment, screening, identifying carcinogenesis, and predicting breast cancer stages. Technologies based on mass spectrometry (MS) are a key element for rapidly evaluating and qualifying a significant number of candidate biomarkers for further construction and validation in breast cancer research [15].

Over the last 20 years, MS-based quantitative proteomics has progressed at a tremendous rate for biomarker development and drug screening. Furthermore, proteomics offers a large number of protein expression profiles that use quantitative techniques to predict and create relationships through bioinformatics tools [16]. Current quantitative differences between samples with and without drug therapy, or between normal and abnormal cells, are useful in determining proteins with upregulation or downregulation and for establishing biologic processes and pathways [17].

In the current drug-discovery process, the numerous MS-based systems of medical, functional, and chemical proteomics will integrate and establish synergies. Throughout the complicated drug discovery scenario, the numerous MS-based proteomic methods go further than the general goal of drug target discovery, allowing researchers to investigate drug–target interaction (selectivity and specificity), drug activity (efficacy, resistance, toxicity), and elucidate the mechanism of action of a drug [18].

This study analysed various biological replicates under the same conditions to investigate the alterations in protein expression using LC-MS. This technique revealed differentially expressed proteins by comparing Doxorubicin (DOX) and Doxorubicin-CaCO_3-_nanoparticles (DOX-Ar-CC-NPs) treated cells.

## 2. Materials and Methods

### 2.1. Chemicals and Reagents

MCF-7 cancer cells were purchased from ATCC Collection (Manassas, VA, USA). Cells were maintained in Dulbecco’s Modified Eagle’s medium with 10% FBS, penicillin and streptomycin (both in 100 µg/mL). All cells were incubated at 37 °C, in a CO_2_ humidified atmosphere. Naphthol Blue Black (Amido Black), CHAPS, DTT, and DOX were purchased from Sigma (St. Louis, MO, USA). Antisera were bought from Santa Cruz Biotechnology (Santa Cruz, CA, USA).

### 2.2. Synthesis of the Ar-CC-NPs, Encapsulation of DOX-Ar-CC-NPs, and In Vitro Release Study

The synthesis procedure, encapsulation of DOX-Ar-CC-NPs, and in vitro release study were parts of a previous study that included the synthesis and the characterisation of synthesised and encapsulated nanoparticles as well as the cytotoxicity of the nanoparticles [19].

### 2.3. Cell Culture

MCF-7 cell line was cultured in DMEM medium as described previously [19]. Then, protein concentration was measured utilising a Bradford Assay Kit (Quick Start™ Bradford Protein Assay, Biorad, Hercules, CA, USA) following the producer’s protocol.

### 2.4. Cellular Uptake Assay

Ar-CC-NPs were synthesised and characterised in our previous study that resulted in nanoparticles having an average diameter of 35.50 nm, 19.3% loading content, 97% encapsulation efficiency, and a surface potential and intensity of 19.1 ± 3.9 mV and 100%, respectively [19].

Cellular uptake of DOX and DOX-Ar-CC-NPs by MCF-7 cancer cell line was examined using fluorescence microscopy. The MCF-7 cancer cell was seeded in 6-well dishes with a cell density of 5 × 10^5^ cells for each well. Then, subsequent 24 h, the media was discarded and substitute with a fresh complete growth medium containing 0.5 µg/mL of DOX or DOX-Ar-CC-NPs (at equal concentrations) for 24, 48, and 72 h at 37 °C. Lysol-ID green dye was used after 30 min of incubation. As a result, the media was thrown away, and the cells were rinsed twice with PBS. Then, the cells were treated for 30 min with 4% paraformaldehyde. The cells were washed with PBS and then loaded on microscope slides, and a fluorescent microscope was used to examine the fluorescent distribution (Nikon ECLIPSE Ti S Japan).

### 2.5. High-Resolution Microscopy of Treated MCF-7 Cancer Cells

TEM was used to examine the high-resolution microscopy of the treated MCF-7 cancer cell line; the cells were harvested at 5 × 10^5^ cells/well in 2 mL. Control cells were treated with a complete growth medium only. After the incubation, the cells were washed with PBS and then harvested. Later, cells were incubated with glutaraldehyde (4%) overnight at 4 °C then fixed for 2 h with osmium tetroxide (1%) at 4 °C. Cells were then washed with sodium cacodylate buffer (0.1 M) three times. Then, gradient of acetone was used to dehydrate the cells. The cells were embedded in a beam capsule with (100%) resin for infiltration and then polymerised at 60 °C for 48 h. Subsequently, the required area was sectioned into ultrathin sections by an ultra-microtome; loaded onto copper grids, and uranyl acetate and citrate were used for staining; then, samples were viewed using High-resolution TEM (H-7100 Hitachi, Tokyo, Japan).

### 2.6. Annexin FITC-V/Propidium Iodide Staining for Apoptosis Determination

The investigation was carried out following the manufacturer’s guidelines (BD Bioscience, San Jose, CA, USA), with minor modifications. Cells were cultured following Section 2.6. Following incubation, the detached cells, in addition to the adherent cells, were discarded. Cells were harvest, centrifuged at 1300× *g*, then MCF-& cells were suspended with 100 µL of 1× phosphate-buffered saline, and 5 µL of Annexin-V FITC was added to the cells. After that, 5 µL of PI solution were added. Samples were then resuspended through delicate pipetting. At 10 min later, cells were transferred to the dark chamber at room temperature; 400 µL of 1× assay buffer and 10 µL of PI (0.5 µg/mL) were added, and specimens were analysed using FACSCalibur flow cytometer (BD Bioscience, San Jose, CA, USA).

### 2.7. Protein Extraction and Quantification

#### 2.7.1. Radioimmunoprecipitation Assay (RIPA) Method

MCF-7 proteins were extracted using RIPA method. RIPA lysis buffer was used to prepare crude lysate (0.1% SDS, 1% NP-40, 20 mM HEPES buffer, 150 mM NaCl, and one tablet of protease inhibitor cocktail). Scraping cells from tissue culture plates was followed by sedimentation to create a cellular pellet. The cell pellets then suspended in RIPA buffer and incubated at 4 °C with constant rotation before being centrifuged at 10,000× *g* for 30 min [20].

#### 2.7.2. Bicinchoninic Acid (BCA) Protein Assay

The MCF-7 crude protein load in the pellet was assessed using the BCA Protein Assay Kit. Fifty parts of BCA (Reagent A) were mixed with 1 part of BCA (Reagent B), according to the manufacturer protocol. A total of 25 µL of each sample and standard were used in the 96-well plate in three replicates, and 200 µL of working reagent was added in the wells that containing both the standard and samples. Then, the plate was incubated for 30 min at 37 °C and 5% CO_2_ min, then cooled to room temperature before reading the absorbance at 570 nm to plot the standard curve [21].

### 2.8. 1-D SDS-Gel (One-Dimensional Polyacrylamide Gel Electrophoresis)

One-dimensional SDS-PAGE was used to detect each extracted protein according to Laemmli. Extracted proteins were diluted (1:1) with sample loading buffer (10% SDS, 0.5 M Tris-HCl, and Bromophenol Blue) to 10 µL then heated at 95 °C for 5 min. The proteins were run using 5% stacking gel and 12% resolving gel at 80 V, using a Mini-Protean system (Biorad Laboratories, Hercules, CA, USA). The molecular weight of the proteins was estimated by loading the protein ladder (Biorad Laboratories, Hercules, CA, USA). Then, Coomassie Brilliant Blue (CBB) was used to stain the gels. CBB staining solution was shaken for 6 h at room temperature; then, the gels were destained by using 40% methanol destaining solution for 40 min [22].

### 2.9. Trypsin Digestion

Each single protein band was excised and destained using a washing solution with 50% Acetonitrile in 100 mM ammonium bicarbonate.

The gel fragments were then reduced in a 60 °C water bath for 30 min with a 100 mM DTT reduction solution. The reduced samples were then incubated in the dark for 20 min with an alkylation solution (55 mM IAA in 100 mM ABC). Samples were washed and incubated for 15 min in 100% acetonitrile (ACN), then samples were digested by trypsin solution (7 µg/µL) and incubated overnight at 30 °C. Finally, the digested peptides were mixed and dried by vacuum centrifuge for 30 min then kept at 80 °C until the next analysis.

### 2.10. Proteomic Analysis Using Orbitrap MS and Statistical Analysis

Proteomic analysis was conducted using Orbitrap mass spectrometer (Thermo Scientific, Waltham, MA, USA). Orbitrap MS spectra had the following parameters: scan (320–1800 *m*/*z*), resolving power of 120,000, automatic gain control (AGC) target of 4.0 and 50 (ms) injection time. The technique used a 3 s top speed mode for a maximum 3 s loop. A monoisotopic *m*/*z* and a precursors charge state of 2–7 were examined further for MS/MS. Then, the precursors were screened using 20 s exclusion and an intensity threshold of 5000. Ion trap MS (ITM) was used to analyse the MS/MS spectra, with the following parameters: a rapid scan rate of and resolving power of 60,000, an AGC target of 1.0 × 10^2^ (100), and a maximum injection time of 250 (ms). Thermo Scientific^TM^ Proteomic Discoverer^TM^ Software Version 2.1 was used for data analysis, using database: *Homo sapiens*, MS2 tolerance: 0.6 Da, missed cleavage: 2, oxidation (M), deamination of asparagine (N) and glutamine (Q), carbamidomethyl (C) based on a 5% false discovery rate (FDR). Statistical analysis in this study conducted using Minitab software16 software (Minitab Inc. state College, PA, and the USA) and Origin 8. Data are presented as mean ± SD (standard deviation). Treatment effects were assessed with an ANOVA test with a significance value of *p* ˂ 0.05 except indicated otherwise [23].

### 2.11. Bioinformatics Analyses

Bioinformatics analyses of the proteomics data were performed using the Ingenuity Pathways Analysis (IPA) software (Ingenuity^®^Systems, http://www.ingenuity.com/) [24] to predict the affected pathways related to the changes of protein abundance in response to the treatment of DOX-Ar-CC-NPs compared to DOX. Details of proteins with their quantitative MS/MS score that indicates their confidence in protein identification were introduced into the IPA software. Each identified protein was mapped and overlaid onto a global molecular network developed from the Ingenuity Knowledge Base. The IPA comparison analysis was performed to compare the similarity and difference among the affected pathways and biological functions in DOX-Ar-CC-NPs- and DOX-treated cells. The heat map was generated to visualise the canonical pathways simultaneously affected by both treatments whose activity appears to increase or decrease as shown by the positive and negative activation z-scores, respectively, that correlate with specific biological functions.

## 3. Results

### 3.1. DOX and DOX-Ar-CC-NPs Cellular Uptake

**Figure 1:**
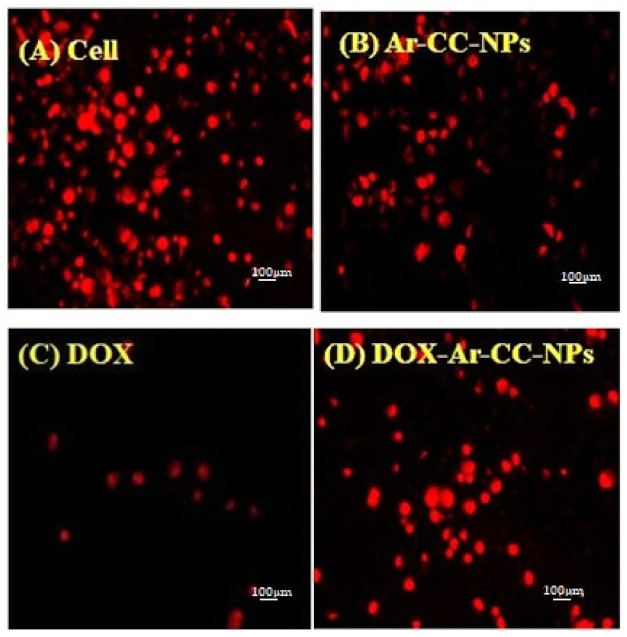
Cellular uptake study. (**A**) Control cells with a red dot showing DOX. (**B**) Cells treated with Ar-CC-NPs. (**C**) Cells treated with DOX. (**D**) Cells treated with DOX-Ar-CC-NPs (×100).

MCF-7 cancer cell lines were treated with DOX and DOX-Ar-CC-NPs for 48 and 72 h. Results in Figure 1 revealed that after 24 h of DOX-Ar-CC-NPs exposure, faint red fluorescence signals were observed in the cytoplasm. The intensity of the red fluorescence was increased in the cytoplasm as the duration increased to 48 h, and strong red fluorescence was detected inside the nucleus after 72 h, which indicates that the DOX was released from the DOX-Ar-CC-NPs and diffused to the nucleus Figure 1D. While free DOX revealed faint red fluorescence inside the nucleus after 24 h, this was improved with increasing the time of exposure to 48 h, as seen in Figure 1C. This result revealed direct DOX interaction with the cells, which strongly induced in the nuclei of MCF-7 cells. The incubation time was found to be positively linked to the cell uptake intensities of both DOX and DOX-Ar-CC-NPs, implying that increasing the incubation time can result in more particles entering cells. As a result, nanoparticles based on DOX-Ar-CC-NPs may aid in the induction of apoptosis.

### 3.2. Ultrastructural Observation of MCF-7 Cells Using TEM

**Figure 2:**
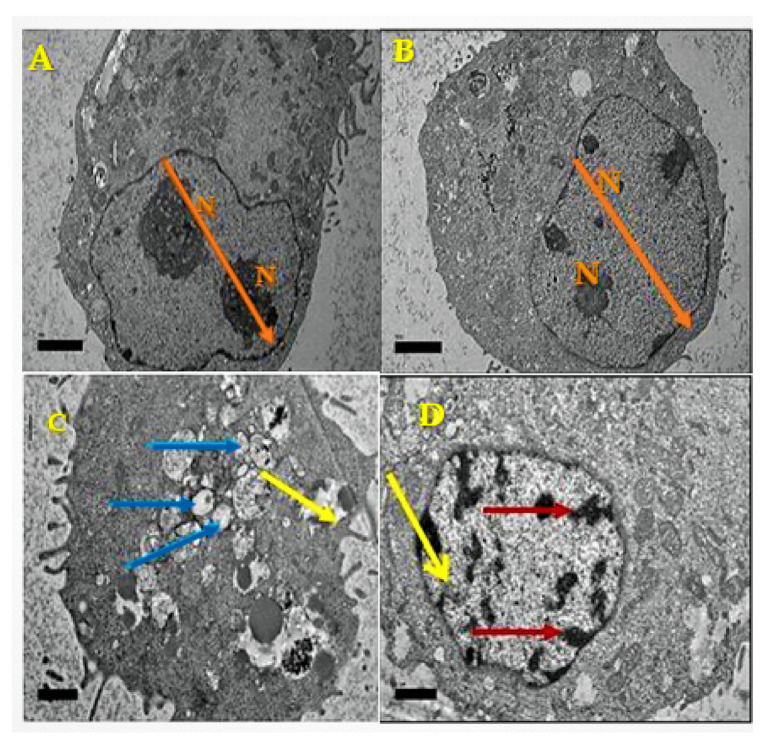
Transmission electron microscope of MCF-7 cancer cells. (**A**) Control (untreated) cells (magnification ×10,000) and nucleus as indicated with N. (**B**) Cells treated with Ar-CC-NPs (magnification ×10,000) and nucleus as indicated with N. (**C**) Cells treated with DOX-Ar-CC-NPs (magnification ×7000) with cell dispersion effect as indicated with blue arrow and membrane blebbing indicated by the yellow arrow. (**D**) Cells treated with DOX-Ar-CC-NPs (magnification ×5000) with chromatin condensation indicated with a yellow arrow, while red arrow indicates DOX-Ar-CC-NPs in the endosome vesicles. Bar 2 µm.

**Figure 3:**
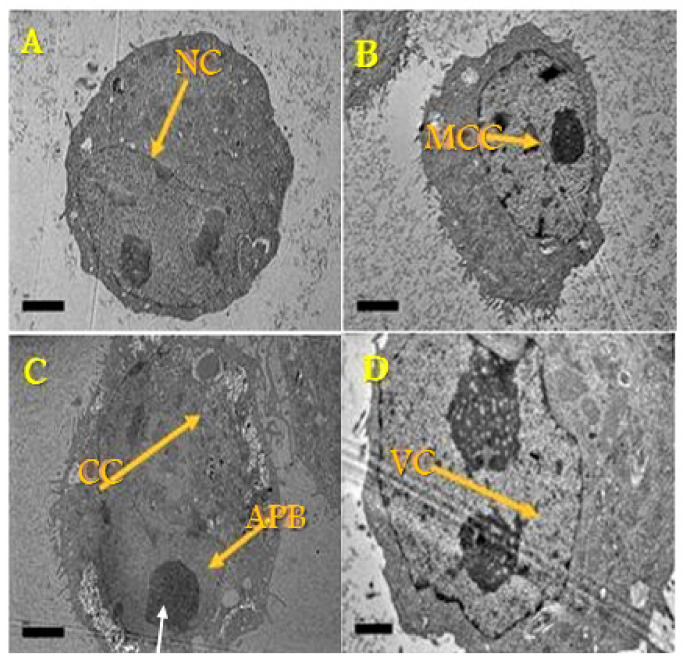
Transmission electron microscope of ultrastructural features of untreated MCF-7 cancer cells. (**A**) Control (untreated) cells with nucleus represented by NC (magnification ×9000). (**B**) Cells treated with Ar-CC-NPs with condensed cristae of mitochondria represented by MCC (magnification ×8000). (**C**) Cells treated with DOX alone will cell dispersion represented by CC and apoptotic body (APB) (magnification ×9000). (**D**) Cells treated with DOX-Ar-CC-NPs with vesicles in the cytoplasm VC (magnification ×15,000).

The cell morphology differences were investigated to assess the effect of DOX-alone and DOX-Ar-CC-NPs on MCF-7 cancer cells using TEM (Figure 2 and Figure 3). The micrographs of control cells and cells treated with Ar-CC-NPs revealed chromatin, clear nuclear membrane (Figure 2A,B). The ultrastructural physical characteristics were notably altered after exposure to DOX (Figure 2C) or DOX-Ar-CC-NPs, demonstrating characteristic apoptotic phenomena. The most important notices of early apoptosis comprising cell shrinkage and merging can be witnessed in both treated groups. Many vesicles precast around the cytoplasm and a group of vacuoles containing DOX-Ar-CC-NPs coacervates were found (Figure 2D). This proved that DOX-Ar-CC-NPs can internalise in cells. Membrane blebbing, as well as apoptotic bodies, were also observed and similar to the results observed in flow cytometry (Figure 3), which confirmed that DOX-Ar-CC-NPs can promote cell death in MCF-7 cancer cells.

### 3.3. Annexin-V FITC Assay of MCF-7 Cancer Cells

**Figure 4:**
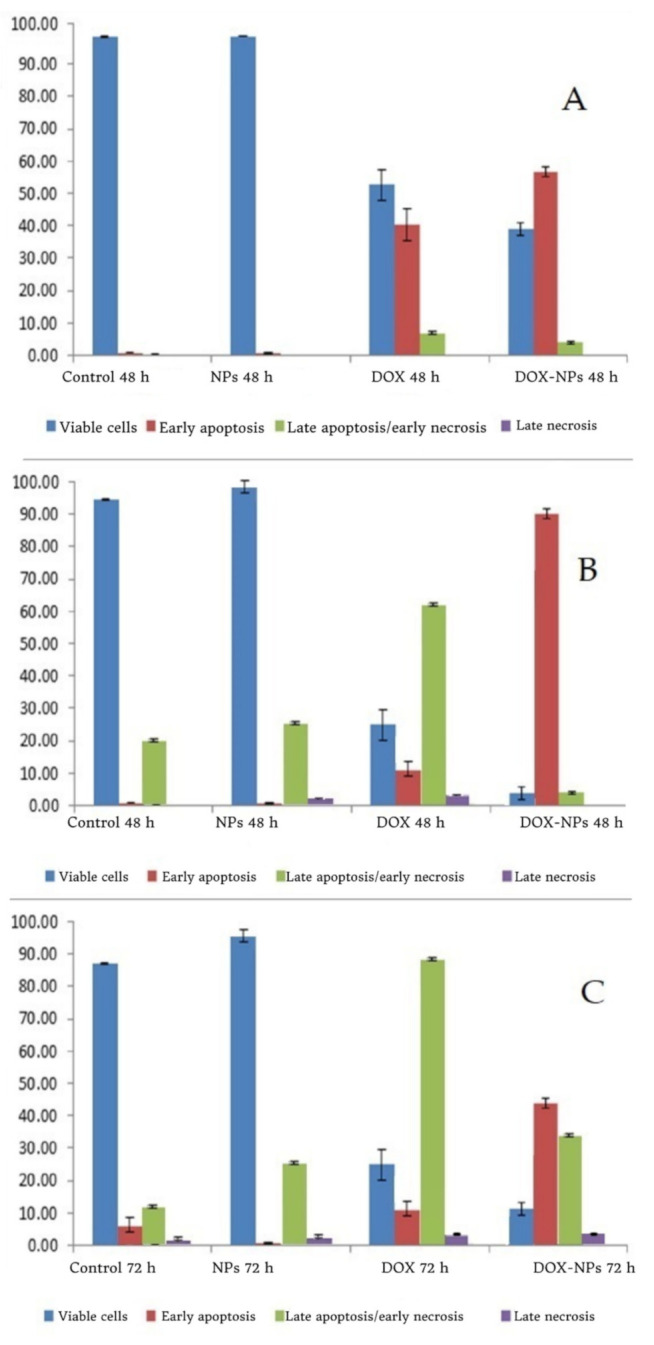
The flow cytometry histograms. (**A**) 24 h of treatment with the control, AR-CC-NPS, DOX, and DOX-Ar-CC-NPs groups. (**B**) 48 h of treatment, with the control, AR-CC-NPS, DOX, and DOX-Ar-CC-NPs groups. (**C**) 72 h of treatment with the control, AR-CC-NPS, DOX, and DOX-Ar-CC-NPs groups.

At 24, 48, and 72 h, the proportions of early apoptotic cells in the DOX-Ar-CC-NPs treatment community were 56.69 ± 1.67%, 96.91± 0.36%, and 50.03 ± 0.46%, respectively, while the proportions of late apoptotic cells were 3.98 ± 0.30%, 2.22 ± 0.24, and 40.34 ± 0.01%, respectively (Figure 4). DOX-Ar-CC-NPs treatment greatly improved the proportion of apoptotic cells in comparison to the non-treated cells (*p* ˃ 0.05). A similar result was shown in the DOX-treated group. Moreover, there was no significant difference between the Ar-CC-NPs treated cells and non-treated cells (*p* ˃ 0.05). DOX and DOX-Ar-CC-NPs can also cause time-dependent MCF-7 cells death.

### 3.4. Annexin V and PI Binding Assay

**Figure 5:**
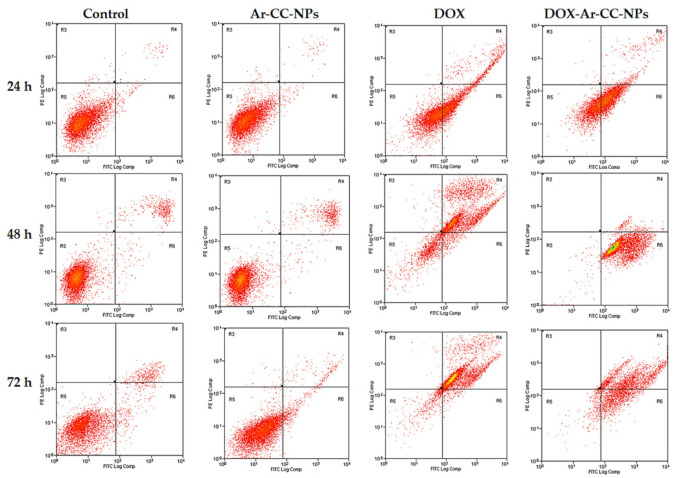
Annexin V assays of MCF-7 cells for 24 h, 48 h, and 72 h, respectively.

The aim of Annexin V and PI binding was to study apoptotic, necrotic, and cell death. The MCF-7 results show a dot plot in the lower right and upper right quadrant populations, indicating early and late apoptotic cells in that region, whereas the lower left quadrant indicates cell viability (Figure 5). Necrotic cells are identified by a population of cells stained only with PI in the upper left quadrant. The control group consisted of cells that had not been exposed to any treatment. The proportion of cell viability in the control group at 24, 48, and 72 h was observed to be 98.46 ± 0.18%, 93.38 ± 0.92%, and 88.83 ± 0.61%, respectively, as seen in Table 1. For 24, 48, and 72 h, the percentage of cell viability in the free Ar-CC-NPs group was 98.54 ± 0.04%, 91.63 ± 1.25%, and 92.86 ± 0.65%, respectively. There was no significant difference between Ar-CC-NPs treated cells and non-treated cells (*p* ˃ 0.05), indicating that Ar-CC-NPs can influence chemo-resistance and drug-targeting delivery, and thus could reduce unwanted side effects. By comparing between control and Ar-CC-NPs treated groups, the levels of both early and late apoptosis in DOX and DOX-Ar-CC-NPs groups are significantly higher. In the DOX-treated group, a similar result was found. When the cells were treated with DOX for 24, 48, and 72 h, the average percentage of Annexin V-staining positive cells (quantity of apoptotic cells) increased significantly from 48.89 to 86.55 and 96.95%, in that order. These findings confirmed that DOX-Ar-CC-NPs could promote apoptosis in MCF-7 cells in a time-dependent manner.

### 3.5. Assessment of Protein Extract Using 1-D-Gel SDS-PAGE

**Figure 6:**
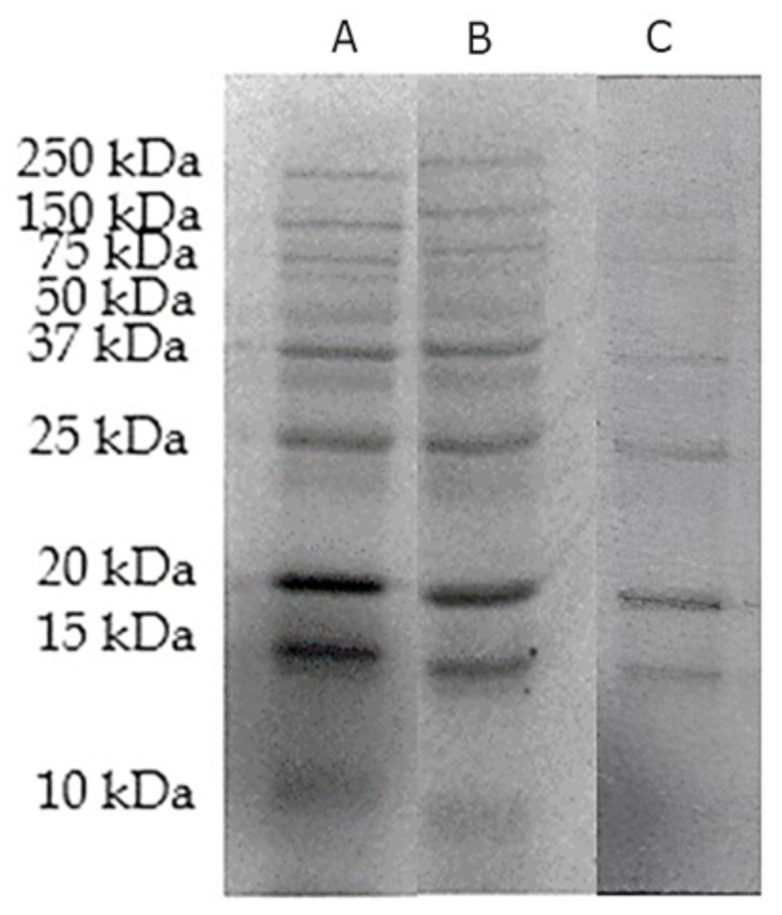
SDS-PAGE for proteins extracted from, (**A**) MCF-7 cancer cell line treated with DOX-Ar-CC-NPs. (**B**) MCF-7 cancer cell line treated with DOX. (**C**) Untreated MCF-7 control group.

Modulation in protein expression was detected using 1D SDS-PAGE according to their molecular weight using Biorad Precision Plus Protein Dual Color Standards between 10 and 250 kDa, (Figure 6).

**Figure 7:**
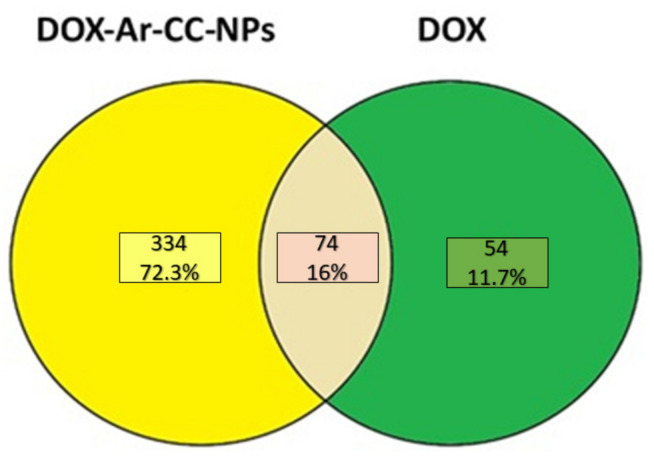
Venn diagram depicting the comparison of proteins identified in DOX-Ar-CC-NPs and DOX-treated MCF-7 cells (breast cancer cells). The overlap represents 74 proteins whose expressions are significantly correlated (*p* < 0.00001) statistical significances differences were estimated by ANOVA.

Proteomic analysis of MCF-7 cancer cell line treated with DOX and DOX-Ar-CC-NPs were evaluated through the LC-MS/MS method. The Venn diagram (Figure 7) shows the number of proteins expressed in both cultures (DOX and DOX-Ar-CC-NPs). A total of 334 proteins have been expressed in DOX-Ar-CC-NPs treated cells, while 54 proteins expressed in only the DOX treatment. Out of the 334 proteins expressed in DOX-Ar-CC-NPs treated cells, 36 protein IDs showed elevation in protein abundance (Table 2), while in DOX-treated cells, it was revealed that there were 7 proteins elevated in abundance out of 54 proteins (Table 3).

### 3.6. Top Canonical Pathways Correlated with Biological Functions Affected by Changes in Protein Abundance in DOX-Ar-CC-NPs- Compared to DOX-Treated Cells

**Figure 8:**
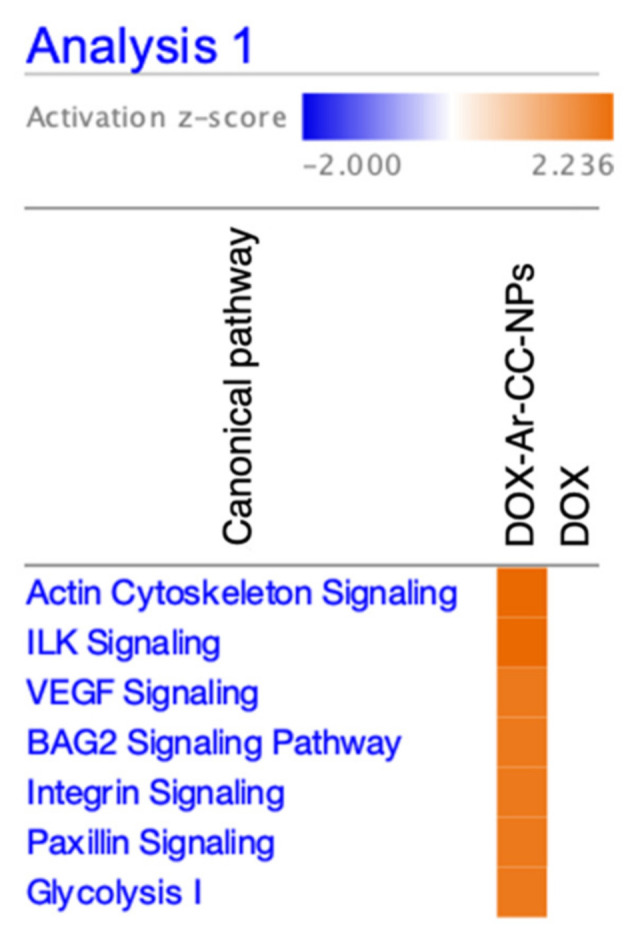
Heat map generated from the IPA comparison analysis showing the top 7 canonical pathways affected by changes of proteins in response to the treatments of DOX-Ar-CC-NPs compared to DOX. Orange and blue bars indicate positive and negative activation z-scores, respectively, while transparent bar indicates no activation.

The IPA comparison analysis between the protein abundance in DOX-Ar-CC-NPs- and DOX-treated cells identified seven canonical pathways affected by the changes of 14 proteins abundance, namely: ACTB, ACTC1, ACTN1, ACTN4, ANXA2, ENO1, ENO3, GAPDH, HSPA5, HSPA8, HSPA9, MYH9, PKM, and VIM. Figure 8 visualises the heat map generated from IPA software showing Actin cytoskeleton signalling and Integrin-linked kinase (ILK) signalling as the two top canonical pathways with the activation z-scores of 2.236. In addition, the other five canonical pathways, Vascular Endothelial Growth Factor (VEGF) signalling, BCL2-associated athanogene 2 (BAG2) signalling, Integrin signalling, Paxillin signalling, and Glycolysis I have the activation z-scores of 2 (Table 4). The scores indicated that all seven pathways were activated in DOX-Ar-CC-NPs treatment (orange bar) but not activated in DOX treatment (transparent bar).

**Figure 9:**
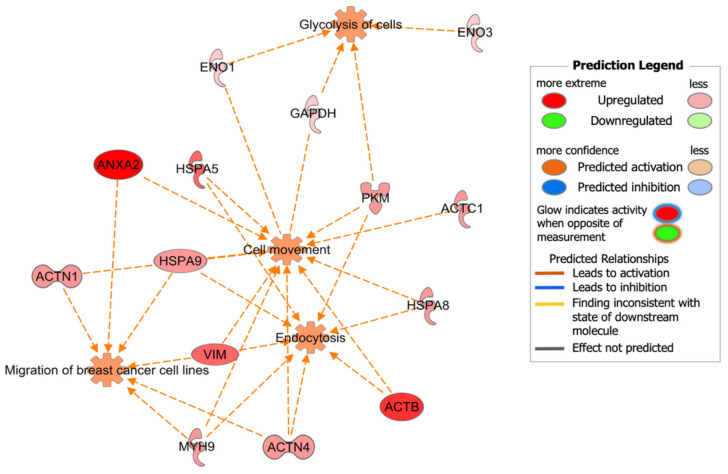
IPA graphical representation of the predicted biological functions linked to the top 7 canonical pathways affected by changes of proteins in response to the treatments of DOX-Ar-CC-NPs compared to DOX. The network is shown as nodes (proteins) and edges (represent biological relationships). Nodes in red indicate represent a high abundance of proteins. Various shapes of the nodes symbolise the functional group of the proteins.

Figure 9 shows a graphical representation of the predicted biological functions linked to the top canonical pathways that are listed in Table 4. Overall, the expression changes in 14 proteins, namely, Actin, cytoplasmic 1 (ACTB), Actin, alpha cardiac muscle 1 (ACTC1), Actinin, alpha 1 (ACTN1), Actinin alpha 4 (ACTN4), Annexin A2 (ANXA2), Alpha-enolase (ENO1), Beta-enolase (ENO3), Glyceraldehyde-3-phosphate dehydrogenase (GAPDH), Epididymis secretory sperm binding protein Li 89n (HSPA5), Epididymis luminal protein 33 (HSPA8), HSPA9 protein (HSPA9), Myosin, heavy polypeptide 9 (MYH9), Pyruvate kinase (PKM), and Vimentin (VIM) were associated with cell movement, endocytosis, migration of breast cancer cell lines, and glycolysis of cells.

## 4. Discussion

Our results showed that DOX-Ar-CC-NPs could trigger apoptosis in MCF-7 cells, indicating that Ar-CC-NPs’ drug delivery has a high potency. Furthermore, a fluorescence microscope analysis of cellular uptake revealed clear co-localisation of DOX-Ar-CC-NPs within endosomes and lysosomes. This demonstrated that DOX-Ar-CC-NPs triggered cell death in MCF-7 cancer cells. DOX-Ar-CC-NPs were found to trigger apoptosis in MCF-7 cancer cells in vitro. Flow cytometry analysis showed that Annexin V, a phagocyte membrane protein with a strong affinity for phosphatidylserine (PS) [25], and a flow cytometry study revealed a decrease in cells viability proportion with the increased duration of exposure to DOX-Ar-CC-NPs for 24 and 48 h lead to apoptotic cells increments, while more cells in the late apoptotic stage have been present after an increased time of exposure of 72 h. Early apoptotic cancer cells could become late apoptotic cells once the plasma membrane becomes permeable [26]. DOX-CC-NP concentrations can activate multiple regulatory mechanisms, resulting in apoptosis or cell death.

As compared to conventional chemotherapeutic drugs, nanoparticle delivery was shown to have a higher loading potential, lower toxicity, and greater drug or biomolecule stability [9]. As a result, this research examined multiple biological replicates under the same conditions to investigate changes in protein expression using LCMS to identify differentially expressed proteins by comparing DOX and DOX-Ar-CC-NPs treated cells.

Among the 30 detected proteins that were differently expressed in DOX-Ar-CC-NPs treated cells, pyruvate kinase, ATP synthase, enolase, glyceraldehyde-3-phosphate dehydrogenase, mitochondrial ADP/ATP carrier, and trypsin are the key enzymes engaged in energy metabolism, suggesting that far more energy must be employed by glycolysis to support tumour cells survive during subjected to chemical stress,. The other identified proteins that are structural include: Keratin, α- and β-tubulin, actin, and actinin. Additionally, one of the heat shock proteins was identified, which is Hsp90; other proteins include annexins and human epididymis protein 4. The proteins identified in DOX treated cells were tubulin alpha-1B chain and a beta chain, actin cytoplasmic 1, annexin A2, IF rod domain-containing protein, and 78 kDa glucose-regulated protein.

### 4.1. Heat Shock Proteins

Breast cancer is one of the leading causes of death in women globally [27]. Hsp90 is an energy-dependent molecular chaperone [28]. According to research, Hsp90 expression levels have been linked to disease development and survival in melanoma non-small cell lung cancer [29]. Furthermore, increased Hsp90 expression has been linked to a reduction in breast cancer survival [30], and inhibiting it can prevent proliferation and improve cell death in breast cancer cells, indicating that Hsp90 could be used as a therapeutic biomarker [31].

### 4.2. Structural Proteins

Keratins (KRTs) are a group of essential proteins found in normal epithelia, but some of them revealed a high expression rate in cancers [32]. KRTs that are differentially expressed enable molecular diagnosis of a variety of cancers, including GI tumours, and KRTs can be used as biological markers to distinguish metastatic from original adenocarcinoma [33]. Additionally, KRTs have previously been found to be epithelial differentiation markers [34].

Different types of KRTs were differentially expressed in DOX-Ar-CC-NPs treated cells. The tubulin superfamily comprises γ-, δ-, ε-, η-families, as well as the α- and β-tubulin families that make up the tubulin dimer. The microtubule nucleation assembly at the centrosome is aided by -tubulins. The functions of other tubulin families are yet to be determined. Nine isoforms of α-tubulin in humans are found, including tubulin α1A, α1B, α1C, α3C, α3D, α3E, α4A, α4B, and α8 [35].

At the moment, the precise roles of the tubulin isoforms are unknown. The differences in sequence and structure between tubulin isoforms are insignificant. Tubulin isoforms’ C-terminal tails are the most changeable domains [36]. Additionally, β-tubulin in humans include nine isoforms [37]. Human BC cells express a variety of tubulin isoforms, including βI-, βII-, βIII-, and βIVB-tubulins [38]. The levels of expression of βII-, βIII-, and βIVB-tubulins have been linked to cancer development [39]. In our study, different types of tubulins were identified, including: alpha-1B chain, tubulin beta-4B chain, and tubulin beta chain.

The dynamic actin cytoskeleton collaborates with E-cadherin- and integrin-based cell–cell or cell-matrix adhesions to preserve the polarised epithelial structure and to produce the force needed for cell form changes and cell migration in remodelling tissues [40]. The regulated cooperation between actin and adhesions sometimes fails in malignant epithelia, leading to the loss of the organisation of polarised epithelial and increase the plasticity of morphological cells that leads cancer cells to invade and spread [41].

The invasive phenotypes of cancer are closely linked to the protein expression of actinin-4. Unlike the other ACTNs, alpha-actinin 4 (ACTN4) has distinct signalling transduction, nuclear translocation, and gene expression regulation. According to early studies, ACTN4 is thought to be a component of the motile system of breast cancer cells and is strongly expressed in the nucleus, suggesting that ACTN4 is involved in breast cancer tumorigenesis emergence. Other evidence in breast cancer and other cancers support this theory. Nevertheless, there is no clear evidence linking the tumorigenic phenotype to ACTN4-mediated pathological mechanisms [42]. These findings agreed with our results that revealed the altered expression of both actinin alpha 1 and alpha 4 in DOX-Ar-CC-NPs treated cells.

### 4.3. Proteins Associated with Carbohydrate and Energy Metabolism

It was indicated that normal cells obtain much of their energy from the Krebs cycle (aerobically), while cancer cells obtain it from glycolysis (anaerobically) [32]. Many research conducted in leading scientific journals recently has revealed that tumour cells sustain an elevated rate of glycolysis [43]; this may be due to enhanced enzyme expression, and as a result, tumour cells consume far more energy in comparison to the normal ones [44,45].

Glycolysis is a key mechanism in glucose degradation that involves the breakdown of glucose into two pyruvates. In the glycolytic pathway, the essential metabolic activity of pyruvate kinase (PK) is to stimulate the transformation of phosphate in phosphoenolpyruvate (PEP) to adenosine diphosphate, creating ATP and Pyruvic acid [46]. Pyruvate kinase is essential for regulating cell metabolism. The transformation of PEP to Pyruvic acid is the final stage of glycolysis. In mammals, tissue-specific forms of Pyruvate Kinase are found, including PKR, PKL, PKM1, and PKM2, with the last two produced by alternative splicing of a single mRNA transcript from the PKM gene. PKM2 oligomers are found in both low-activity dimers and high-activity tetramers. PKM2 controls the rate-limiting stage of glycolysis in tumour cells, which changes energy metabolism from respiratory consumption to lactate formation. Aside from its function as a metabolic controller, it also functions as a protein kinase, which aids in tumorigenesis [47].

In many cases, metabolic changes primarily entail high glucose uptake and glycolysis, resulting in the production of ATP and lactic acid in the cytosol. Although mitochondria are functional, this activity is called the Warburg effect [48]. In fast-developing tumours where hypoxia occurs instead, increased glycolysis can fuel a sufficient amount of ATP production [49]. ATP synthase is one of the main mitochondrial enzymes, which generates ATP and provides energy by inducing phosphorylation of ADP through a transmembrane proton gradient [50]. In our study, both ATP synthase subunit alpha and beta were identified.

Enolase-1 is an endothelial cell-derived oxidative stress protein that has an important role in the glycolytic pathway’s conversion of 2-phosphoenolpyruvic acid. During hypoxia, changes in enolase-1 abundance and other enzymes in the glycolytic pathway causes tumour cells to respond to the energy demand, which enhances survival and proliferation [51], and enhance the aggressive and advanced ability of the cells [52].

Glyceraldehyde-3-phosphate dehydrogenase (GAPDH) is involved in a group of cellular activities, including metabolism and gene transcription [23]. GAPDH is the main enzyme in the glucose metabolism that is involved in oxidation reduction by transforming glyceraldeh2yde-3-phosphate to 1,3-bisphosphoglycerate by reducing NAD+ to NADH [53]. Changes in GAPDH abundance is related to the development and aggressiveness of breast cancer [54].

One of the most common proteins in the mitochondria inner membrane is the ADP/ATP transporter. In eukaryotic cells, the carrier’s function provides the transport steps in oxidative phosphorylation since it introduces the spent fuel ADP back into the mitochondrion for ATP synthesis and exports the synthesised ATP to the cytosol [43]. The energy carrier is a part of the organelle carrier family of movement proteins, which includes many other carriers that exchange different metabolites in and out of those organelles [55,56].

Trypsin, a well-known digestive serine protease, has also been linked to cancer and has been shown to improve proliferation, invasion, and metastasis [43].

### 4.4. Other Proteins

Annexins are a class of proteins that bind phospholipids in the calcium (2+)-dependent method. Many Annexins are involved in tumour development. The ANXA3 protein is linked to reduced disease development in breast cancer patients and plays a role in apoptosis regulation in vitro by altering the Bcl-2/Bax balance [57].

Human epididymis protein 4 (HE4) is a protein that has a conserved motif and has been identified in a variety of protease inhibitors [58]. HE4 was originally believed to be involved in the non-specific mechanism response of several epithelia but has now been discovered to also play a role in epithelial host defence [59].

As compared to natural tissue, HE4 is particularly overexpressed in epithelial ovarian cancer (EOC) [60]. Clinically, HE4 has been described as a novel therapeutic biomarker for EOC, as well as effective in the diagnosis of recurrent disease [61]. HE4 expression is linked to lymph node involvement and may be a risk factor for breast cancer.

As compared to natural tissue, HE4 is particularly showed a different abundance in ovarian tissue in epithelial ovarian cancer [60]. Previously, HE4 has been identified as a therapeutic biomarker for EOC and has also shown useful in the detection of recurrent disease [61]. HE4 expression is associated with lymph node involvement and is a possible predictive factor of breast cancer recurrence [62]. HE4 may serve as a cancer biomarker for the early detection of breast cancer [63].

### 4.5. Bioinformatics Analyses Revealed That the Abundance of Proteins Affected the Migration of Cells and Glycolysis

Bioinformatics analyses revealed that all six top canonical pathways, including the signallings of Actin cytoskeleton, ILK, VEGF, BAG2, Integrin, and Paxillin, are associated with the migration of breast cancer cells. Actin polymerisation induced the extracellular matrix (ECM) remodelling to form membrane protrusions that increased the breast cancer cells motility and invasion [64]. ILK mediates integrin signalling by connecting the cytoplasmic domains of β-integrins to the actin cytoskeleton to regulate the migration of breast cancer, and thus promotes cell survival [65]. Moreover, VEGF regulates the networks of proteins to mediate the filopodia formation in the membrane, which consequently increase the ability of breast cancer cell migration [66]. Besides that, a high abundance of BAG2 regulates the pro-cathepsin B/annexin II complex formation and facilitates the secretion of pro-cathepsin B, which induce metastasis in breast cancer [67]. As a scaffolding protein, paxillin has been shown to control the signalling of estrogen to induce PI3K complex signalling to support breast cancer cell attachment, migration, and invasion [68]. On the other hand, accumulating evidence suggests that aerobic glycolysis (the Warburg effect) is a crucial metabolic adaptation of cancer cells to predominantly utilise glycolysis for energy production despite having a low abundance of oxygen [69]. Targeting glycolysis pathways may provide a possible drug for breast cancer therapy.

Hence, these comparison analyses showed that the treatments of DOX-Ar-CC-NPs might induce the control and regulation of breast cancer cells migration and endocytosis, as well as aerobic glycolysis.

## 5. Conclusions

Our results indicate that DOX-Ar-CC-NPs can trigger cell death in MCF-7 cells, indicating the high potency of Ar-CC-NPs in drug delivery. Furthermore, cellular uptake experiments using a fluorescence microscope revealed clear co-localisation of DOX-Ar-CC-NPs inside endosomes and lysosomes. This indicated that DOX-Ar-CC-NPs triggers apoptosis of MCF-7 cancer cells in vivo. The quantitative estimate of apoptosis induction was confirmed further by the Annexin V/PI apoptosis flow cytometry study, which showed that DOX-Ar-CC-NPs trigger apoptosis in MCF-7 cells. Furthermore, this study shows that proteomic study is an effective technique for examining protein expression correlated with chemotherapy drugs on cancerous cells; this approach opens the door to identifying therapeutic targets for the treatment of MCF-7 cells, and the LC-MS/MS system enabled the identification of a group of proteins in the two-dimensional gel study, as well as proteins with larger pIs and high molecular weight.

## Figures and Tables

**Table 1:** Annexin V/(PI) apoptosis assay of MCF-7 cancer cells for 24, 48 and 72 h treatment in different groups. The left lower (LL) quadrant represents viable cells, Left-Right (LR) Quadrant represent early apoptosis, Under Right (UR) Quadrant represent late apoptosis while the Under Lower (UL).

Time		Control	Ar-CC-NPs	DOX	DOX-Ar-CC-NPs
	LL	98.46 ± 0.18	98.54 ± 0.04	52.71 ± 4.78	39.22 ± 1.94
24 h	LR	0.83 ± 0.08	0.68 ± 0.07	40.30 ± 5.08	56.69 ± 1.67
	UR	0.48 ± 0.05	0.45 ± 0.04	6.95 ± 0.32	3.98 ± 0.30
	UL	0.24 ± 0.04	0.34 ± 0.06	0.05 ± 0.01	0.14 ± 0.06
	LL	93.38 ± 0.92	91.63 ± 1.25	22.19 ± 2.83	0.87 ± 0.14
48 h	LR	0.71 ± 0.14	0.65 ± 0.03	8.36 ± 2.05	96.91 ± 0.36
	UR	5.74 ± 0.77	7.57 ± 1.15	67.92 ± 4.18	2.22 ± 0.24
	UL	0.17 ± 0.06	0.21 ± 0.10	1.53 ± 0.67	0.00 ± 0.00
	LL	88.83 ± 0.61	92.86 ± 0.65	8.90 ± 2.24	8.61 ± 0.42
	LR	2.11 ± 0.32	3.99 ± 1.14	3.55 ± 0.66	50.03 ± 0.46
72 h	UR	8.53 ± 0.69	2.05 ± 0.40	85.70 ± 2.80	40.34 ± 0.01
	UL	0.71 ± 0.21	0.37 ± 0.06	1.86 ± 0.08	1.03 ± 0.03

**Table 2:** Characteristics of identified proteins in MCF-7 cancer cells treated with DOX-Ar-CC-NPs using 1DE.

No.	Protein Name	Taxonomy	Accession No. ^a^	Score ^b^	Matches ^c^	Coverage % ^d^	MW ^e^	PI ^f^	Th. MW ^g^	Th. PI ^h^
1.	Actin, cytoplasmic 1	*Homo sapiens* (Human)	P60709	315.1	88	71.2	41.71	5.48	1841.25	5.54
2.	Glucose-regulated protein	*Homo sapiens* (Human)	V9HWB4	277.8	80	50.3	72.288	5.16	5088.28	4.33
3.	Chaperonin	*Homo sapiens* (Human)	A0A024R3X4	247.1	69	40.1	61.016	5.87	4747.86	4.33
4.	Annexin A2	*Homo sapiens* (Human)	P07355	335.7	88	57.5	38.58	7.75	856.09	4.00
5.	Vimentin OS = Homo sapiens	*Homo sapiens* (Human)	P08670	235.6	76	40.9	53.619	5.12	2699.14	4.51
6.	Actinin, alpha 1, isoform CRA_a	*Homo sapiens* (Human)	A0A024R694	134.3	43	30.3	102.993	5.41	2699.14	4.51
7.	Epididymis luminal protein 33	*Homo sapiens* (Human)	V9HW22	144.4	44	41.1	70.854	5.52	2949.33	3.92
8.	ATP synthase	*Homo sapiens* (Human)	V9HW31	172.7	51	48.2	56.525	5.4	2423.32	4.51
9.	Myosin, isoform CRA_a	*Homo sapiens* (Human)	A0A024R1N1	124	41	12.5	226.392	5.6	5546.36	4.57
10.	Pyruvate kinase	*Homo sapiens* (Human)	V9HWB8	171	56	33.1	57.9	7.84	1627.68	6.56
11.	Actinin alpha 4 isoform 1 (Fragment)	*Homo sapiens* (Human)	A0A0S2Z3G9	115.4	41	30.8	104.788	5.44	3253.85	8.30
12.	Elongation factor 1-alpha (Fragment)	*Homo sapiens* (Human)	Q53G85	121.8	42	48.9	50.081	9.01	3518.07	6.84
13.	HSPA9 protein (Fragment)	*Homo sapiens* (Human)	Q8N1C8	138.3	44	31.1	73.808	6.37	2265.53	6.76
14.	Tubulin alpha-1B chain	*Homo sapiens* (Human)	P68363	110.5	31	39.6	50.12	5.06	2017.12	6.61
15.	Actin, alpha cardiac muscle 1	*Homo sapiens* (Human)	P68032	153.1	50	36.8	41.992	5.39	2456.77	5.32
16.	ATP synthase subunit alpha, mitochondrial	*Homo sapiens* (Human)	P25705	147.4	39	30.3	59.714	9.13	4240.06	6.27
17.	Trypsin	*Homo sapiens* (Human)	P07477	167.5	62	31.6	24.394	7.18	3061.55	7.89
18.	Tubulin β chain	*Homo sapiens* (Human)	P68371	113	39	46.7	49.799	4.89	1943.50	5.21
19.	Tubulin β chain	*Homo sapiens* (Human)	P07437	113.2	39	46.3	49.639	4.89	1828.85	5.21
20.	α-enolase	*Homo sapiens* (Human)	A0A2R8Y6G6	94.8	24	23.9	47.297	6.99	1388.54	4.51
21.	Calreticulin, isoform CRA_b	*Homo sapiens* (Human)	V9HW88	79	25	31.8	48.112	4.44	4719.06	7.98
22.	Glyceraldehyde-3-phosphate dehydrogenase	*Homo sapiens* (Human)	P04406	72	20	35.2	36.03	8.46	4447.65	4.39
23.	HSP 90kDa α (Cytosolic), isoform CRA_a	*Homo sapiens* (Human)	A0A024RD80	74.6	26	22.3	83.212	5.03	7038.38	7.85
24.	cDNA FLJ53906, highly similar to Tubulin beta chain	*Homo sapiens* (Human)	B7Z4N1	62.8	24	40.3	39.869	4.97		
25.	Histone H2A	*Homo sapiens* (Human)	A0A0U1RRH7	72.4	17	33.5	18.541	11.5	1145.24	5.98
26.	ADP, ATP carrier protein	*Homo sapiens* (Human)	Q59EI9	61.4	18	24.1	35.361	9.85	5857.86	11.16
27.	Beta-enolase	*Homo sapiens* (Human)	P13929	69.5	17	15.8	46.957	7.71	1314.92	3.67
28.	Epididymis luminal protein 4	*Homo sapiens* (Human)	D0PNI1	62.1	19	34.6	27.728	4.79	2949.33	3.92
29.	Actin-like protein (Fragment)	*Homo sapiens* (Human)	Q562L5	61.7	20	85.4	11.518	6.35	2859.31	6.26
30.	cDNA FLJ54371, highly similar to Serum albumin	*Homo sapiens* (Human)	B4DPP6	63.6	20	12.4	70.317	6.09		
31.	Keratin, type I cytoskeletal 10	*Homo sapiens* (Human)	P13645	469.9	127	55.3	58.79	5.2	2911.32	4.95
32.	Keratin, type I cytoskeletal 9	*Homo sapiens* (Human)	P35527	379.7	110	58.5	62.03	5.2	2911.32	4.95
33.	Keratin, type II cytoskeletal 2 epidermal	*Homo sapiens* (Human)	P35908	315.1	100	58.3	65.39	8	4079.69	4.55
34.	Keratin 1	*Homo sapiens* (Human)	H6VRG1	389.1	115	44.8	66.09	8.1	830.94	8.75
35.	Keratin, type II cytoskeletal 5	*Homo sapiens* (Human)	P13647	73.5	26	16.10	62.34	7.7	3024.48	4.95
36.	Keratin, type I cytoskeletal 14	*Homo sapiens* (Human)	P02533	60.02	21	23.7	51.5	5.16	2911.32	4.95

^a^ Uniprot database accession number. ^b^ Score: MS/MS search score. ^c^ Number of peptides matches. ^d^ Coverage: matching peptide amino acid coverage of. ^e^ Experimental molecular weight (kDa). ^f^ Experimental PI. ^g^ Theoretical molecular weight (kDa) from ExPASy database. ^h^ Theoretical pI from ExPASy database.

**Table 3:** Characteristics of identified proteins in MCF-7 cancer cells treated with DOX using 1DE.

No.	Protein	Taxonomy	Accession No. ^a^	Score ^b^	Matches ^c^	Coverage % ^d^	MW ^e^	PI ^f^	Th. MW ^g^	Th. PI ^h^
1.	78 kDa glucose-regulated protein	*Homo sapiens* (Human)	V9HWB4	64.7	23	20.4	72.288	5.16	3179.27	4.36
2.	Annexin A2	*Homo sapiens* (Human)	P07355	96.7	30	32.1	38.58	7.75	856.09	4.00
3.	Putative keratin-87 protein	*Homo sapiens* (Human)	A6NCN2	76	28	41.9	29.099	5.8	2640.88	6.65
4.	Tubulin beta chain	*Homo sapiens* (Human)	P07437	69.2	25	36.9	49.639	4.89	1828.85	5.21
5.	IF rod domain-containing protein	*Homo sapiens* (Human)	A0A140TA62	122.9	44	19.2	49.38	5.06	3655.25	4.56
6.	Tubulin alpha-1B chain	*Homo sapiens* (Human)	P68363	61.7	22	33.4	50.12	5.06	2017.12	6.61
7.	Actin, cytoplasmic 1	*Homo sapiens* (Human)	P60709	71.8	27	34.9	41.71	5.48	1841.25	5.54

^a^ Uniprot database accession number. ^b^ Score: MS/MS search score. ^c^ Number of peptides matches. ^d^ Coverage: matching peptide amino acid coverage of. ^e^ Experimental molecular weight (kDa). ^f^ Experimental PI. ^g^ Theoretical molecular weight (kDa) from ExPASy database. ^h^ Theoretical pI from ExPASy database.

**Table 4:** IPA analysis. A summary of IPA comparison analysis showed the top predicted canonical pathways affected by changes of proteins in response to the treatments of DOX-Ar-CC-NPs compared to DOX.

Top Canonical Pathway	Treatment	
**Actin cytoskeleton signalling**	**DOX-Ar-CC-NPs**	**DOX**
*p* value	2.95 × 10^−5^	6.68 × 10^−2^
Activation z-score	2.236	N. A
Genes	ACTB, ACTC1, ACTN1, ACTN4, MYH9	ACTB
**Integrin-linked kinase (ILK) signalling**	**DOX-Ar-CC-NPs**	**DOX**
*p* value	4.19 × 10^−7^	5.4 × 10^−2^
Activation z-score	2.236	N. A
Genes	ACTB, ACTC1, ACTN1, ACTN4, MYH9, VIM	ACTB
**VEGF signalling**	**DOX-Ar-CC-NPs**	**DOX**
*p* value	1.4 × 10^−5^	2.74 × 10^−2^
Activation z-score	2	N. A
Genes	ACTB, ACTC1, ACTN1, ACTN4, MYH9	ACTB
**BAG2 signalling**	**DOX-Ar-CC-NPs**	**DOX**
*p* value	6.85 × 10^−6^	2.19 × 10^−4^
Activation z-score	2	N. A
Genes	ANXA2, HSPA5, HSPA8, HSPA9	ANXA2, HSPA5
**Integrin signalling**	**DOX-Ar-CC-NPs**	**DOX**
*p* value	2.58 × 10^−4^	5.73 × 10^−2^
Activation z-score	2	N. A
Genes	ACTB, ACTC1, ACTN1, ACTN4	ACTB
**Paxilin signalling**	**DOX-Ar-CC-NPs**	**DOX**
*p* value	1.99 × 10^−5^	3.00 × 10^−2^
Activation z-score	2	N. A
Genes	ACTB, ACTC1, ACTN1, ACTN4	ACTB
**Glycolysis I**	**DOX-Ar-CC-NPs**	**DOX**
*p* value	1.99 × 10^−5^	-
Activation z-score	2	-
Genes	GAPDH, ENO1, ENO3, PKM	-

## Data Availability

Not applicable.
